# A new goodness‐of‐fit measure for probit models: Surrogate *R* ^2^

**DOI:** 10.1111/bmsp.12289

**Published:** 2022-10-17

**Authors:** Dungang Liu, Xiaorui Zhu, Brandon Greenwell, Zewei Lin

**Affiliations:** ^1^ Department of Operations, Business Analytics and Information Systems University of Cincinnati Carl H. Lindner College of Business Cincinnati Ohio USA; ^2^ Department of Business Analytics and Technology Management College of Business and Economics, Towson University Towson Maryland USA

**Keywords:** categorical data, model comparison, OLS *R* ^2^, probit analysis, pseudo *R* ^2^, surrogate residual

## Abstract

Probit models are used extensively for inferential purposes in the social sciences as discrete data are prevalent in a vast body of social studies. Among many accompanying model inference problems, a critical question remains unsettled: how to develop a goodness‐of‐fit measure that resembles the ordinary least square (OLS) *R* ^2^ used for linear models. Such a measure has long been sought to achieve ‘comparability’ of different empirical models across multiple samples addressing similar social questions. To this end, we propose a novel *R* ^2^ measure for probit models using the notion of surrogacy – simulating a continuous variable S as a *surrogate* of the original discrete response (Liu & Zhang, Journal of the American Statistical Association, 113, 845 and 2018). The proposed *R* ^2^ is the proportion of the variance of the surrogate response explained by explanatory variables through a *linear model*, and we call it a surrogate *R* ^2^. This paper shows both theoretically and numerically that the surrogate *R* ^2^ approximates the OLS *R* ^2^ based on the latent continuous variable, preserves the interpretation of explained variation, and maintains monotonicity between nested models. As no other pseudo *R* ^2^, McKelvey and Zavoina's and McFadden's included, can meet all the three criteria simultaneously, our measure fills this crucial void in probit model inference.

## INTRODUCTION

Probit models are used widely in social research to make inference as binary and ordinal outcomes are common in economic, political, behavioural, and psychological studies. Broadly speaking, there are three basic inference problems that need to be solved in probit analysis: (i) Which variables are significant for explanatory analysis? (ii) Which variables should be included for predictive analysis? (iii) Given the variables selected in (i) or (ii) and a candidate model, how well does the model fit the data? Problems (i) and (ii) can be tackled using the likelihood ratio test (hypothesis testing) and the Akaike (AIC) or Bayesian (BIC) information criteria (variable selection), respectively. However, the hypothesis testing or variable selection solutions do not address problem (iii): how well does the selected model fit the data?

A solution for problem (iii), such as an overall goodness‐of‐fit measure, is critical in empirical research. When linear models are employed, for instance, the ordinary least squares (OLS) *R* ^2^ is one of the key inference outcomes empirical researchers rely on in model assessment. It indicates the overall explanatory power of a model. Let us consider a situation where a few candidate models are selected using different criteria (e.g., AIC, BIC or lasso). Often these models are comparable and which model is the ‘best’ depends on the evaluation criterion domain experts would like to adopt. The choice of the final model may not be a purely statistical issue when it comes down to only a few candidate models as the difference between them may not be significant. What domain experts are more interested in knowing is what is the explanatory power of these similar models, for example, at the 7% level or 70% level. As the power being 7% or 70% will give completely different insights into the subject matter, it is imperative to have a sensible goodness‐of‐fit measure for probit analysis. For example, if previous studies give *R* ^2^ values all around 70% while the current study yields an *R* ^2^ of 20%, special attention needs to be paid to the design and quality of the study itself.

Given the importance of the notion of *R* ^2^, we have seen unceasing efforts to develop an analogous notion for generalized linear models where the response variable is binary or ordinal (see, for example, Agresti, 1986; Cox & Wermuth, 1992; Cragg & Uhler, 1970; Efron, 1978; Hu et al., 2006; Laitila, 1993; McFadden, 1973; McKelvey & Zavoina, 1975; Zheng & Agresti, 2000). Nevertheless, as pointed out by Hagle and Mitchell (1992) and Veall and Zimmermann (1996) in their survey papers, one of the shortcomings of probit analysis is the lack of an *R* ^2^ statistic that is analogous to the OLS *R* ^2^. Such a shortcoming may prevent domain researchers from comparing empirical models trained from different samples. As an example of this ‘comparability’, consider a study of job satisfaction where data may be collected in three different ways using a quantitative score on a scale of 0–100, a dichotomous yes/no indicator, or a five‐category rating ranging from ‘extremely unsatisfied’ to ‘extremely satisfied’. Although neither the samples nor the empirical models used to draw inferences are the same, ‘most empirical researchers are explicitly or implicitly making rough comparisons of “goodness of fit” across’ these models and samples, because they address similar domain questions (Veall & Zimmermann, 1996). In social studies, ‘the research experience in the area is far more important than any statistical criteria’ such as what specific method is used to select variables (Veall & Zimmermann, 1996). It is therefore vital to have a goodness‐of‐fit measure for probit models that is analogous to the OLS *R* ^2^, as it will ensure comparability of different models across different samples for similar research questions.

### A motivating counterexample of the established McKelvey–Zavoina *R* ^2^

Among the goodness‐of‐fit measures developed so far for probit models, McKelvey and Zavoina's (1975) *R* ^2^ is perhaps the most commonly used pseudo *R* ^2^ (Hagle & Mitchell, 1992; Simonetti et al., 2017; Veall & Zimmermann, 1996). It is recommended by several review papers (Hagle & Mitchell, 1992; Veall & Zimmermann, 1996; Windmeijer, 1995), as it may be the only one that carries the interpretation of explained variation and it best approximates the OLS *R* ^2^ if the underlying continuous response can be observed.

Consider using the following probit model to fit a binary or ordinal response:

$$\mathrm{Pr}\left\{Y\leq j\right\}=\Phi \left\{\alpha_{j}-\left(\beta_{1}X_{1}+\cdots +\beta_{q}X_{q}\right)\right\},\;j=1,\ldots ,J,$$

where J≥2 is the number of discrete categories, and $\Phi \left(\cdot \right)$ is the cumulative distribution function of the standard normal distribution. Assuming the existence of a latent continuous outcome Z, McKelvey and Zavoina (1975) define a goodness‐of‐fit measure

$$R_{\mathrm{MZ}}^{2}=\frac{\sum_{i=1}^{n}{\left({\hat{z}}_{i}-\hat{\bar{z}}\right)}^{2}}{\sum_{i=1}^{n}{\left({\hat{z}}_{i}-\hat{\bar{z}}\right)}^{2}+n},$$

where ${\hat{z}}_{i}=x_{1i}{\hat{\beta }}_{1}+\cdots +x_{q,i}{\hat{\beta }}_{q}$ and $\hat{\bar{z}}=\sum_{i=1}^{n}{\hat{z}}_{i}/n$. The denominator represents the total variation on the scale of the latent variable, and the nominator the variation explained by $X_{1},\ldots ,X_{q}$. This is the only goodness‐of‐fit measure that has the explained‐variation interpretation, among others surveyed by Hagle and Mitchell (1992) and Veall and Zimmermann (1996). Both survey papers advocated the use of $R_{\mathrm{MZ}}^{2}$ by further showing with numerical evidence that among existing goodness‐of‐fit measures, $R_{\mathrm{MZ}}^{2}$ best approaches the OLS *R* ^2^ if the latent outcome Z can be observed. Nevertheless, our counterexample shows a remarkable discrepancy between $R_{\mathrm{MZ}}^{2}$ and the OLS *R* ^2^ in a simple setting. In fact, the example proves that $R_{\mathrm{MZ}}^{2}$ does not maintain *monotonicity* between nested models.

Example 1 (non‐monotonicity of $R_{\mathrm{MZ}}^{2}$). We simulate 50,000 observations from the probit model for a binary response

$$\mathrm{Pr}\left(Y=1|X\right)=\Phi \left(\beta_{0}+\beta_{1}X_{1}+\beta_{2}X_{2}\right),$$

where $\beta ={\left(\beta_{0},\beta_{1},\beta_{2}\right)}^{Τ}={\left(2,-1,0.7\right)}^{Τ}$, $X_{1}∼U\left(-3,3\right)$, $X_{2}=X_{1}1_{X_{1}\leq -0.2}+\left(2.8-X_{1}\right)1_{X_{1}>-0.2}$ such that $X_{2}∼U\left(-3,3\right)$, and the correlation $\rho_{X_{1},X_{2}}\approx .70$. We calculate the *R* ^2^ measures for the full model which includes both $X_{1}$ and $X_{2}$ and a reduced model which contains $X_{1}$ only. Displayed in Table 1 are the ordinary least squares $R_{\mathrm{OLS}}^{2}$, McKelvey and Zavoina's $R_{\mathrm{MZ}}^{2}$, and our surrogate $R_{S}^{2}$. The unobservable $R_{\mathrm{OLS}}^{2}$ serves as the benchmark in the comparison. When both of the explanatory variables are included, the three *R* ^2^ measures are close to each other (=.61). If we drop $X_{2}$ from the model, both $R_{\mathrm{OLS}}^{2}$ and $R_{S}^{2}$ naturally fall to a smaller value of .31. A shocking observation is that McKelvey and Zavoina's $R_{\mathrm{MZ}}^{2}$ moves in a different direction: it *increases* by a sizeable margin and becomes .76.

**TABLE 1:** *R* ^2^ values for the full and reduced models in Example 1

	$R^{2}\left\{X_{1},X_{2}\right\}$	$R^{2}\left\{X_{1}\right\}$	$R^{2}\left\{X_{1},X_{2}\right\}-R^{2}\left\{X_{1}\right\}$
OLS	.61	.31	.30
McKelvey–Zavoina	.61	**.76**	**−.15**
Surrogate	.61	.31	.30

*Note*: Bold values is to highlight numerical results worth attention.

This counterexample proves that McKelvey and Zavoina's $R_{\mathrm{MZ}}^{2}$ cannot guarantee the monotonicity between nested models, which is a key feature of the OLS *R* ^2^. The breakdown of monotonicity may result in a notable difference between $R_{\mathrm{MZ}}^{2}$ and $R_{\mathrm{OLS}}^{2}$. Unfortunately, this serious defect of $R_{\mathrm{MZ}}^{2}$ has been overlooked in the literature ever since it was proposed in 1973. It brings into question the claim, made by previous survey papers, that $R_{\mathrm{MZ}}^{2}$ performs consistently well in terms of approximating $R_{\mathrm{OLS}}^{2}$.

### Criteria for a goodness‐of‐fit measure analogous to the OLS *R* ^2^

The discussion so far has manifested the need for a new goodness‐of‐fit measure for probit models. Similarly to Hagle and Mitchell (1992) and Veall and Zimmermann (1996), we adopt the following criteria to evaluate an *R* ^2^ measure:

(C1) It approximates the OLS *R* ^2^ based on the latent continuous variable Z.

(C2) It preserves the interpretation of explained variation.

(C3) It maintains monotonicity between nested models.

No existing pseudo‐*R* ^2^ measures satisfy criteria (C1)–(C3) simultaneously. For example, McKelvey and Zavoina's $R_{\mathrm{MZ}}^{2}$ does not guarantee that criteria (C1) and (C3) are met, as demonstrated in our counterexample. There is another broad class of pseudo‐*R* ^2^ measures developed based on the ratio of likelihoods; see McFadden's (1973) *R* ^2^, for example. As they measure uncertainty through entropy instead of variance, they do not meet criteria (C1) and (C2) (see our discussion on their merits in other aspects).

In this paper we propose a new notion of the goodness‐of‐fit measure for probit models. We call it a surrogate *R* ^2^. The general idea is to simulate a continuous variable and use it as *a surrogate for the original discrete response*. This novel way of analysing discrete data has been proved useful in solving some previously unsolvable problems; see, for example, Liu and Zhang (2018), Liu et al. (2021), Cheng et al. (2021), and R packages developed by Greenwell et al. (2018) and Li et al. (2021). This paper will show that the general surrogate idea can give rise to a simple yet desirable solution to goodness‐of‐fit measures as well. Specifically, we define a surrogate *R* ^2^ as the proportion of the variance of the *surrogate response* explained by the regressors through a *linear model*. This paper will justify, both theoretically and numerically, that the surrogate *R* ^2^ meets all three criteria (C1)–(C3). It is therefore the only goodness‐of‐fit measure that resembles the OLS *R* ^2^, has the explained‐variation interpretation, and maintains monotonicity between nested models. This property is achieved by generating a common surrogate response from the full model and using it for calculating *R* ^2^ values for all smaller models.

## MEASURING GOODNESS OF FIT BY A SURROGATE *R* ^2^

Suppose a binary or ordinal variable Y has J categories $\left\{1,2,\ldots ,J\right\}$, with the order 1<2<⋯<J. Given a set of explanatory variables, the full model is

(1) $$\mathrm{Pr}\left\{Y\leq j\right\}=\Phi \left\{\alpha_{j}-\left(\beta_{1}X_{1}+\cdots +\beta_{p}X_{p}\right)\right\},\;j=1,\ldots ,J,$$

where $-\infty <\alpha_{1}<\cdots <\alpha_{J}=+\infty$ and some of the coefficients can be zero. The generic symbol $X_{l}$ can represent a single variable X of interest, a high‐order term of X (e.g., $X^{2}$), or an interaction term between X and another variable. It is well known that model (1) can be expressed equivalently through a latent variable:

$$Z=\alpha_{1}+\beta_{1}X_{1}+\cdots +\beta_{p}X_{p}+\varepsilon ,\;\varepsilon ∼N\left(0,1\right).$$

The discrete response Y can be viewed as a censored outcome following

$$Y=\left\{\begin{array}{ll} 1, & \;\text{if}-\infty <Z\leq \alpha_{1}+\alpha_{1},\\ 2, & \;\text{if}\;\alpha_{1}+\alpha_{1}<Z\leq \alpha_{2}+\alpha_{1},\\ \cdots & \\ J, & \;\text{if}\;\alpha_{J-1}+\alpha_{1}<Z<+\infty . \end{array}\right.$$

Different from Liu and Zhang (2018) and Liu et al. (2021), we have written the latent variable Z in such a way that its model contains an intercept term $\alpha_{1}$, and as a result, the cutpoints to obtain Y are $\left(\alpha_{1}+\alpha_{1},\alpha_{2}+\alpha_{1},\ldots ,\alpha_{J-1}+\alpha_{1}\right)$. This mathematical manoeuvre is solely for the purpose of facilitating the calculation of our *R* ^2^. As such, the model for the surrogate variable defined later will also have an intercept term, and there is no need to force it to be zero when implementing our method in statistical software.

For discrete data analysis the surrogate idea is to simulate a continuous response variable and use it as a surrogate of the original discrete response. See Liu and Zhang (2018) and Liu et al. (2021) for a detailed discussion on its application to ordinal data. In the presence of a latent structure, a surrogate response variable can be generated as

$$S∼\left\{\begin{array}{cc} Z|-\infty <Z\leq \alpha_{1}+\alpha_{1}, & \;\text{if}\;Y=1,\\ Z|\alpha_{1}+\alpha_{1}<Z\leq \alpha_{2}+\alpha_{1}, & \;\text{if}\;Y=2,\\ \;\cdots & \\ Z|\alpha_{J-1}+\alpha_{1}<Z<+\infty , & \;\text{if}\;Y=J. \end{array}\right.$$

The variable S has the following properties. (i) S follows the same distribution as the latent variable Z. (ii) Both S and Z yield the same discrete outcome Y, that is, $\alpha_{j-1}+\alpha_{1}<Z\leq \alpha_{j}+\alpha_{1}\Leftrightarrow Y=j\Leftrightarrow \alpha_{j-1}+\alpha_{1}<S\leq \alpha_{j}+\alpha_{1}\;\text{for}\;\mathrm{any}$ *j*. (iii) S is observable, whereas Z is not. These properties provide the theoretical grounds for the use of S as a surrogate response in our analysis and inference, in lieu of the original discrete response Y. This idea will prove useful in developing a new way to measure the goodness of fit of probit models.

Let e denote the surrogate residual variable: $e=S-E\left(S\right)$ (Liu & Zhang, 2018). We can show that

(2) $$S=\alpha_{1}+\beta_{1}X_{1}+\cdots +\beta_{p}X_{p}+e,\;e∼N\left(0,1\right).$$

As such the surrogate residual e is independent of all the explanatory variables $X_{l}$; this is a crucial fact which will be used in Section Resemblance to the OLS *R* ^2^. The relationship between the latent variable Z, the discrete variable Y, and the surrogate variable S can be illustrated below

(3) $$Z\;\left(\text{unobservable}\right)⇀Y\;\left(\text{observed}\right)⇌S\;\left(\text{observable}\right).$$

The arrow ⇀ is read as ‘generates’. For example, Z⇀Y means that Z generates Y. Since S is generated from Y by definition and at the same time the value of S determines the value of Y, we have the relationship Y⇌S. In view of this three‐way relationship, we propose to use the OLS *R* ^2^ of the linear model (2) as a surrogate goodness‐of‐fit measure for the original probit model (1). For notational clarity, we write

$$R_{\left(S\right)}^{2}\left\{X_{1},\ldots ,X_{p}\right\}=\text{the}\;\mathrm{OLS}\;R^{2}\;\text{of the linear model regressing}\;S\;\mathrm{on}\;X_{1},\ldots ,X_{p}.$$

If $\left\{X_{1},\ldots ,X_{q}\right\}$ is a subset of $\left\{X_{1},\ldots ,X_{p}\right\}$, then $R_{\left(S\right)}^{2}\left\{X_{1},\ldots ,X_{q}\right\}$ is calculated using the same surrogate response S generated from the full model (1).

The surrogate *R* ^2^ defined as such inherits the interpretation of explained variation, and it therefore satisfies criterion (C2) set out in the introduction. It is the proportion of the variance of the surrogate response S that can be explained by the covariates. Here, we have used the simulated continuous variable S to substitute the original discrete data Y. Such a substitution is a statistical tactic that can help achieve a close approximation to the OLS *R* ^2^ based on the latent variable, which will be demonstrated in the sections that follow. Furthermore, ‘mapping’ the variability of the ordinal data onto a continuous scale, which is the same scale as the latent variable, also permits the ‘comparability’ among different models and samples as discussed in the introduction. It allows domain experts to compare the goodness‐of‐fit measure of a probit model with that of a linear model, if both samples address the same subject matter. The comparability is in fact part of the motivation of seeking an *R* ^2^ measure for probit models that resembles the OLS *R* ^2^ in social studies.

In the next section, we will show theoretically that the surrogate *R* ^2^ approximates the OLS *R* ^2^ and maintains the monotonicity between nested models. It therefore meets the other two criteria (C1) and (C3) set out in the introduction.

## RESEMBLANCE TO THE OLS *R* ^2^

To see the connection between our surrogate $R_{\left(S\right)}^{2}$ and the OLS *R* ^2^, we review some well‐known results when a sample $z={\left(z_{1},\ldots ,z_{n}\right)}^{\prime }$ of the latent Z can be observed. Let $1_{n\times 1}={\left(1,\ldots ,1\right)}^{’}$, $x_{l}={\left(x_{l1,}\ldots ,x_{\ln}\right)}^{\prime }$, $X=\left(x_{1}|x_{2}|\ldots |x_{p}\right)$, $\bar{x}$ be the 1×p vector of column means of X, and $X_{0}=X-1\bar{x}$. The z‐sample‐based least squares estimates are

$${\hat{\beta }}^{\left(z\right)}={\left(X_{0}^{\prime }X_{0}\right)}^{-1}X_{0}^{\prime }z\;\text{and}\;{\hat{\alpha }}_{1}^{\left(z\right)}=\bar{z}-\bar{x}{\hat{\beta }}^{\left(z\right)}.$$

The fitted values are given by $\hat{z}=1{\hat{\alpha }}_{1}^{\left(z\right)}+X{\hat{\beta }}^{\left(z\right)}$. *R* ^2^ can be expressed as the ratio of the regression sum of squares (SSR) and the total sum of squares (SST):

$$R_{\mathrm{OLS}}^{2}=\frac{{\mathrm{SSR}}_{\left(Z\right)}}{{\mathrm{SST}}_{\left(Z\right)}}=\frac{∥\hat{z}-1\bar{z}{∥}^{2}}{∥z-1\bar{z}{∥}^{2}}=\frac{∥\hat{z}-1\bar{z}{∥}^{2}}{∥\hat{z}-1\bar{z}{∥}^{2}+∥z-\hat{z}{∥}^{2}}=\frac{∥X_{0}{\hat{\beta }}^{\left(z\right)}{∥}^{2}}{∥X_{0}{\hat{\beta }}^{\left(z\right)}{∥}^{2}+∥P_{V_{x}^{⊥}}z{∥}^{2}}.$$

where the linear space $V_{x}=ℒ\left(1,x_{1},\ldots ,x_{p}\right)$, and $P_{V_{x}}$ is the projection to $V_{x}$. It is known that the following results hold: (i) ${\hat{\beta }}^{\left(z\right)}∼N\left(\beta ,{\left(X_{0}^{’}X_{0}\right)}^{-1}\right)$; (ii) $∥P_{V_{x}^{⊥}}z{∥}^{2}∼\chi_{\left(n-p-1\right)}^{2}$; and (iii) ${\hat{\beta }}^{\left(z\right)}$ and $∥P_{V_{x}^{⊥}}z{∥}^{2}$ are independent (Stapleton, 1995, p. 81).

Using a sample of the surrogate response $s={\left(s_{1},\ldots ,s_{n}\right)}^{’}$, *R* ^2^ is given by

$$R_{\left(S\right)}^{2}=\frac{{\mathrm{SSR}}_{\left(S\right)}}{{\mathrm{SST}}_{\left(S\right)}}=\frac{∥\hat{s}-1\bar{s}{∥}^{2}}{∥s-1\bar{s}{∥}^{2}}=\frac{∥X_{0}{\hat{\beta }}^{\left(s\right)}{∥}^{2}}{∥X_{0}{\hat{\beta }}^{\left(s\right)}{∥}^{2}+∥P_{V_{x}^{⊥}}s{∥}^{2}},$$

where ${\hat{\beta }}^{\left(s\right)}={\left(X_{0}^{’}X_{0}\right)}^{-1}X_{0}^{’}s$ is the s‐sample‐based least squares estimate. Similarly to z‐based inference, the surrogate‐response‐based inference satisfies: (i') ${\hat{\beta }}^{\left(s\right)}∼N\left(\beta ,{\left(X_{0}^{’}X_{0}\right)}^{-1}\right)$; (ii') $∥P_{V_{x}^{⊥}}s{∥}^{2}∼\chi_{\left(n-p-1\right)}^{2}$; and (iii') ${\hat{\beta }}^{\left(s\right)}$ and $∥P_{V_{x}^{⊥}}s{∥}^{2}$ are independent.

In view of results (i)–(iii) for z‐based inference and (i')‐(iii') for s‐based inference, we can immediately establish the following result.

Proposition 1 *Given a fixed design matrix* X *with a fixed sample size* n *, the surrogate* $R_{\left(S\right)}^{2}$ *follows the same distribution as* $R_{\mathrm{OLS}}^{2}$.

As the sample size n→∞, it is known that, in probability, ${\hat{\beta }}^{\left(z\right)}\to \beta$, ${\hat{\beta }}^{\left(s\right)}\to \beta$, $∥P_{V_{x}^{⊥}}z{∥}^{2}/\left(n-p-1\right)\to 1$, and $∥P_{V_{x}^{⊥}}s{∥}^{2}/\left(n-p-1\right)\to 1$. Let $Q_{x,n}=X_{0}^{\prime }X_{0}/\left(n-1\right)$ and assume $Q_{x}=\lim_{n\to \infty }Q_{x,n}$ exists. Then both $R_{\left(S\right)}^{2}$ and $R_{\mathrm{OLS}}^{2}$ converge in probability to

$$\frac{\beta^{\prime }Q_{x}\beta }{\beta^{\prime }Q_{x}\beta +1}.$$

Given a random X, the matrix $Q_{x,n}$ converges to $\sum_{x}=\mathrm{Cov}\left(X\right)$, the variance–covariance matrix of X, under some weak conditions. Thus, we immediately have the following result, which states that $R_{\left(S\right)}^{2}$ and $R_{\mathrm{OLS}}^{2}$ approaches the same limiting value.

Proposition 2 If the explanatory variables $X_{l}$ are random, then both $R_{\left(S\right)}^{2}$ and $R_{\mathrm{OLS}}^{2}$ converge to $\frac{\beta^{\prime }\sum_{x}\beta }{\beta^{\prime }\sum_{x}\beta +1}$ in probability, where $\sum_{x}$ is the variance–covariance matrix of **X**.

**FIGURE 1:**
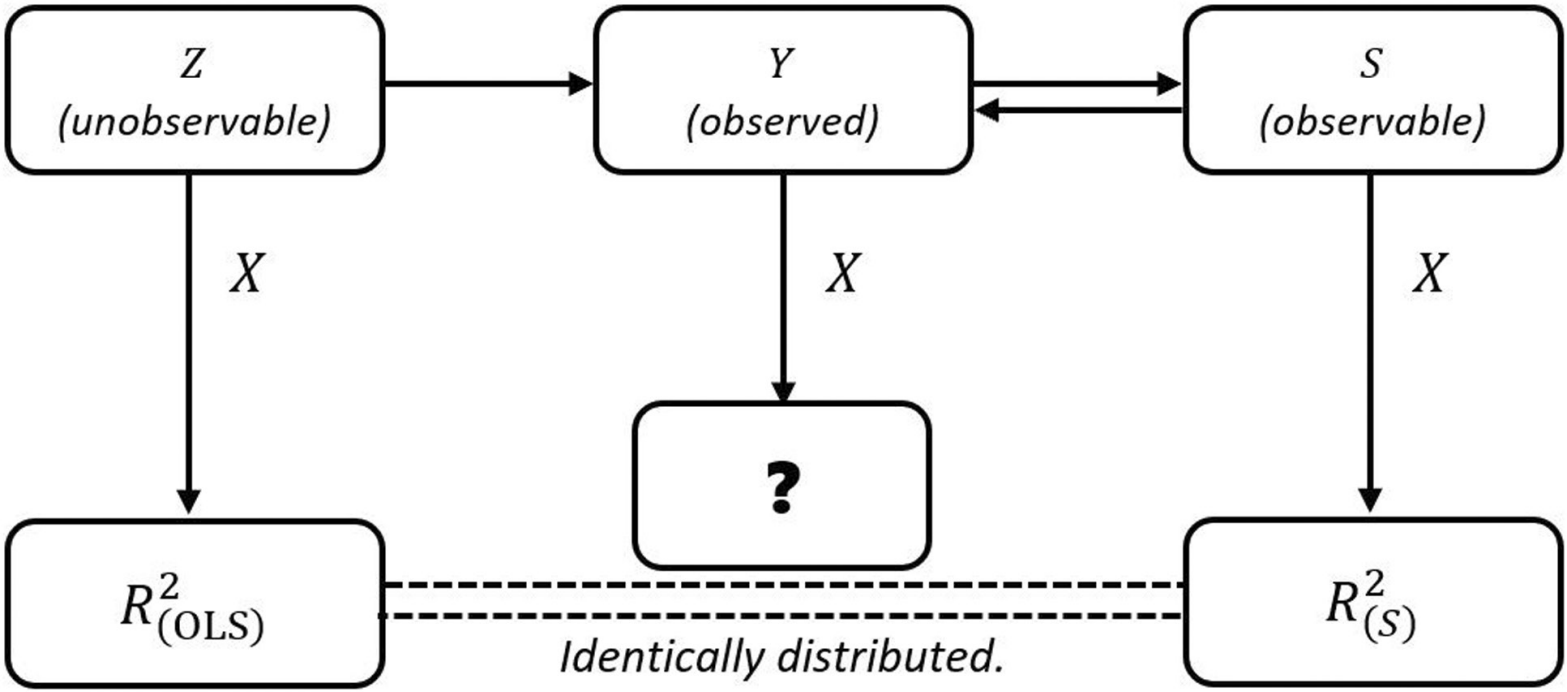
An illustration of the ‘sandwich rule’ that supports the use of $R_{\left(S\right)}^{2}$ as a goodness‐of‐fit measure for the probit model with a discrete response Y.

Propositions 1 and 2 have shown that the surrogate $R_{\left(S\right)}^{2}$ resembles the OLS *R* ^2^ in the sense that, probabilistically, the former behaves in the same way as the latter. In light of these results, we illustrate in Figure 1 the relationship between the two *R* ^2^ measures. The diagram advocates the use of the surrogate *R* ^2^ to measure the goodness of fit of probit models as we observe that (i) the discrete data of Y are ‘sandwiched’ by continuous data of Z and S; and (ii) the z‐based and s‐based *R* ^2^ measures are the ‘same’ in the sense that they follow the same distribution, at least asymptotically. The sandwich rule may suggest we use either $R_{\mathrm{OLS}}^{2}$ or $R_{\left(S\right)}^{2}$ as a measure of how well the model fits the data. As the former is not available to us in practice, our only choice is to use $R_{\left(S\right)}^{2}$.

It is known that for linear models, the OLS *R* ^2^ is equal to the square of the correlation coefficient between the actual and fitted values of the response variable. There is also a one‐to‐one correspondence between $R_{\mathrm{OLS}}^{2}$ and the F statistic. In what follows we will show that similar results can be established for our surrogate $R_{\left(S\right)}^{2}$.

Proposition 3 (Equivalence to the correlation coefficient between actual and fitted values). *The surrogate* $R_{\left(S\right)}^{2}\equiv r_{s\hat{s}}^{2}$ *, where* $r_{\cdot \cdot }$ *is the sample Pearson correlation coefficient and* $\hat{s}=1{\hat{\alpha }}_{1}^{\left(s\right)}+X{\hat{\beta }}^{\left(s\right)}$.

The proof is straightforward given that $s-1\bar{s}=\left(s-\hat{s}\right)+\left(\hat{s}-1\bar{s}\right)$ and $\left(s-\hat{s}\right)⊥\left(\hat{s}-1\bar{s}\right)$. The sample Pearson correlation coefficient between s and $\hat{s}$ is

$$r_{s\hat{s}}=\frac{\left(s-1\bar{s},\hat{s}-1\bar{s}\right)}{∥s-1\bar{s}∥∥\hat{s}-1\bar{s}∥}=\frac{∥\hat{s}-1\bar{s}{∥}^{2}}{∥s-1\bar{s}∥∥\hat{s}-1\bar{s}∥}=\frac{∥\hat{s}-1\bar{s}∥}{∥s-1\bar{s}∥}=\sqrt{R_{S}^{2}}.$$

To see the connection of our $R_{\left(S\right)}^{2}$ to the F statistic, we define

$$F_{\left(S\right)}=\frac{∥\hat{s}-1\bar{s}{∥}^{2}/p}{∥s-\hat{s}{∥}^{2}/\left(n-p-1\right)}.$$

The standard regression theory guarantees that the statistic $F_{\left(S\right)}$ should follow an F distribution with p and n−p−1 degrees of freedom, if the regression coefficients in the probit model (1) satisfy that $\beta_{1}=\cdots =\beta_{p}=0$. As a simple algebraic derivation yields

$$F_{\left(S\right)}=\frac{\left(n-p-1\right)R_{\left(S\right)}^{2}}{p\left(1-R_{\left(S\right)}^{2}\right)},$$

we can establish a one‐to‐one relation between the F statistic and $R_{\left(S\right)}^{2}$.

Proposition 4 (Correspondence to the *F* statistic). *The surrogate R‐squared has a one‐to‐one correspondence to the* F *statistic through*

$$R_{\left(S\right)}^{2}=\frac{{\mathrm{pF}}_{\left(S\right)}}{{\mathrm{pF}}_{\left(S\right)}+\left(n-p-1\right)}.$$

The purpose of presenting Propositions 3 and 4 is to show that the surrogate *R* ^2^ is analogous to the OLS *R* ^2^. We are certainly not interested in this paper in predicting the surrogate response or carrying out hypothesis testing. Finally, borrowing the theory for linear models, we can show that the surrogate $R_{\left(S\right)}^{2}$ maintains monotonicity between nested models.

Proposition 5 (Monotonicity). *If* $\left\{X_{1},\ldots ,X_{q1}\right\}$ *is a subset of* $\left\{X_{1},\ldots ,X_{q2}\right\}$ *, then their surrogate R* ^2^ *measures maintain the order* $R_{\left(S\right)}^{2}\left\{X_{1},\ldots ,X_{q1}\right\}\leq R_{\left(S\right)}^{2}\left\{X_{1},\ldots ,X_{q2}\right\}.$

To guarantee monotonicity, we require that (i) a common surrogate response S be generated using a full model in (1); and (ii) this surrogate response be used to calculate $R_{\left(S\right)}^{2}$ for all smaller models. We offer detailed discussions on this point in the last section.

The exact distributions of $R_{\left(S\right)}^{2}$ depend on the regression coefficients β in the full model (1), which is used to generate the surrogate response S. As long as the estimates of the β s are consistent, the distributional statements in this section still hold, albeit in an approximate sense. The closeness of the approximation will be evaluated in the simulation study in Section Simulation studies.

## INFERENCE BY MULTIPLE SAMPLING

Our proposed *R* ^2^ measure relies on the simulated surrogate response S. It should be acknowledged that any one‐time simulation‐based statistic carries another layer of uncertainty. To reduce this uncertainty to the extent that the simulation has little impact on decision‐making, we can consider performing multiple sampling as suggested by Liu et al. (2021). For example, let $S_{\left(m\right)}$ denote the surrogate response based on the m th simulation conditional on the data $\left(y,x\right)$, and $R_{S_{\left(m\right)}}^{2}$ its corresponding surrogate *R* ^2^. We can use the average ${{\bar{R}}^{2}}_{\mathrm{avg},M}=\sum_{m=1}^{M}R_{S_{\left(m\right)}}^{2}/M$ as a ‘stabilized’ point measure. Following the suggestion in Liu et al. (2021), we use M=30 in our numerical implementation.

Algorithm 1 Computing an interval *R* ^2^ measure using the surrogate method, given a sample $\left(y,x\right)$ of size n and a confidence level $100\left(1-\alpha \right)\%$_._

Step 1: To obtain the *b*th bootstrap copy $R_{\left(S_{\left(b\right)}^{*}\right)}^{2}$ of the surrogate *R* ^2^, we.

1. Resample a sample $\left(y_{\left(b\right)}^{*},x_{\left(b\right)}^{*}\right)$ of size n from the given sample $\left(y,x\right)$;
2. Fit model (1) to the bootstrap sample $\left(y_{\left(b\right)}^{*},x_{\left(b\right)}^{*}\right)$ and obtain a bootstrap estimate ${\hat{\beta }}_{\left(b\right)}^{*}$ of the regression coefficient β;
3. Simulate a surrogate response $S_{\left(b\right)}^{*}$ conditional on $\left(y_{\left(b\right)}^{*},x_{\left(b\right)}^{*},{\hat{\beta }}_{\left(b\right)}^{*}\right)$ and obtain a size‐*n* sample $s_{\left(b\right)}^{*}$;
4. Fit a linear regression model to the bootstrap data $\left(s_{\left(b\right)}^{*},x_{\left(b\right)}^{*}\right)$ and calculate its OLS *R* ^2^ measure. This value is denoted by $R_{\left(S_{\left(b\right)}^{*}\right)}^{2}$.

End For loop.

Step 2: The interval measure is ${\mathrm{CI}}_{1-\alpha }=\left(q_{\alpha /2},q_{1-\alpha /2}\right)$,

where $q_{\nu }$ is the νth quantile of the bootstrap copies $\left\{R_{\left(S_{\left(1\right)}^{*}\right)}^{2},\ldots ,R_{\left(S_{\left(B\right)}^{*}\right)}^{2}\right\}$.

Another inference outcome of interest in goodness‐of‐fit assessments is the interval measure. Instead of merely using a single value, it provides a range of the measure with the designated confidence level. In Algorithm 1, we propose a bootstrap‐based pseudo algorithm for constructing such an interval measure. Basically, in step 1, we simply apply our proposed method to the *b*th bootstrap sample $\left(y_{\left(b\right)}^{*},X_{\left(b\right)}^{*}\right)$, which produces the *b*th copy of our surrogate $R_{\left(S_{\left(b\right)}^{*}\right)}^{2}$. This procedure is repeated B times, resulting in a set of bootstrap copies $\left\{R_{\left(S_{\left(1\right)}^{*}\right)}^{2},\ldots ,R_{\left(S_{\left(B\right)}^{*}\right)}^{2}\right\}$. In step 2, the α/2 and 1−α/2 quantiles of this bootstrap set are extracted to form a $100\left(1-\alpha \right)\%$ confidence interval. We use B=2000 in our numerical implementation following the suggestion made by Efron and Tibshirani (1993). As the bootstrap method in essence is a multiple sampling procedure, the derived interval measure has already accounted for the uncertainty in simulating the surrogate response. When the number of resamplings is as large as B=2000, the randomness induced in simulating the surrogate response is unlikely to affect the final interval to a noticeable extent. In the next section, we examine the numerical performance of the proposed interval *R* ^2^ measure. The result shows that our method consistently outperforms McKelvey and Zavoina's method.

## SIMULATION STUDIES

Simulation studies are conducted to numerically examine the point and interval *R* ^2^ measures. We consider settings with a varying number of explanatory variables (p=4,10,20) and varying degrees of correlations (mild, moderate, and strong). We compare our surrogate $R_{S_{\hat{\beta }}}^{2}$ and $R_{S_{\beta }}^{2}$, where the surrogate response S is generated conditional on an estimate $\hat{\beta }$ and the true value of β, respectively; the OLS *R* ^2^ which serves as the benchmark; McKelvey and Zavoina's $R_{\mathrm{MZ}}^{2}$; and McFadden's $R_{\mathrm{McF}}^{2}$ (McFadden, 1973). The inclusion of $R_{\mathrm{McF}}^{2}$ is merely for the sake of completeness as it does not aim to approximate the OLS *R* ^2^. We also include the rate of classification accuracy (or area under the curve [AUC] for binary outcomes) for reference.

**Setting 1** (p=10 with mild correlations). We simulate an ordinal outcome from the probit model (1). Specifically, we set $\left\{\alpha_{1},\alpha_{2}\right\}=\left\{1,2.9\right\}$ such that Y has three categories. Among p=10 explanatory variables, five have non‐zero coefficients {*β*_1_,…,*β*_5_} = {.8, .7, .6, .6, .5} and the rest have zero coefficients. The explanatory variables follow a multivariate normal distribution with marginals $X_{l}∼N\left(0,1\right)$ and Cor(*X*_*l*_, *X*_*m*_) = .2. The setting is illustrated below with the true signals shaded.


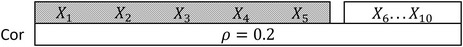


Table 2 presents the four *R* ^2^ measures for the models with different sets of explanatory variables. The numeric values are arithmetic averages over 2000 simulation repetitions. For the full model containing all *X*s, we observe that $R_{\left(S_{\beta }\right)}^{2}$ is very close to $R_{\mathrm{OLS}}^{2}=.799$, even when the sample size is merely 200. If implemented with the estimated parameters, the resulting $R_{\left(S_{\hat{\beta }}\right)}^{2}=.815$ remains close to $R_{\mathrm{OLS}}^{2}$. The numerical difference between the two decreases further, being <1%, as the sample size increases to n=500.

**TABLE 2:** *R* ^2^ measures for probit models used in simulation setting 1

	Full model	Models w/o non‐signals		Models w/o true signals	
	$ℳ\left\{X_{1}\ldots X_{10}\right\}$	$ℳ\left\{-X_{10}\right\}$	$ℳ\left\{-\left(X_{6}\ldots X_{10}\right)\right\}$	$ℳ\left\{-X_{1}\right\}$	$ℳ\left\{-\left(X_{1},X_{2}\right)\right\}$
n=200					
$R_{\mathrm{OLS}}^{2}$	.798	.797	.793	.680	.573
$R_{\left(S_{\beta }\right)}^{2}$	.799	.798	.794	.681	.573
$R_{\left(S_{\hat{\beta }}\right)}^{2}$	.815	.813	.802	.695	.586
$R_{\mathrm{MZ}}^{2}$	.810	.808	.800	.691	.583
$R_{\mathrm{McF}}^{2}$	.496	.493	.483	.374	.289
Accuracy	.745	.744	.739	.677	.625
n=500					
$R_{\mathrm{OLS}}^{2}$	.793	.792	.790	.676	.569
$R_{\left(S_{\beta }\right)}^{2}$	.793	.792	.790	.676	.569
$R_{\left(S_{\hat{\beta }}\right)}^{2}$	.799	.798	.794	.681	.574
$R_{\mathrm{MZ}}^{2}$	.797	.796	.793	.680	.573
$R_{\mathrm{McF}}^{2}$	.479	.478	.474	.362	.281
Accuracy	.735	.735	.733	.669	.620

*Note*: Results presented are arithmetic averages over 2000 simulation repetitions. w/o = without.

When a single non‐signal or all the non‐signals are removed from the model, we calculate *R* ^2^ measures (see the two columns labelled ‘Models w/o non‐signals’). We see a slight reduction in both $R_{\left(S_{\hat{\beta }}\right)}^{2}$ and $R_{\mathrm{OLS}}^{2}$. This is consistent with our expectation as the variables $X_{6},\ldots ,X_{10}$ are not associated with Y. When true signals are removed from the model (see the last two columns in Table 2), a substantial reduction is observed in both $R_{\left(S_{\hat{\beta }}\right)}^{2}$ and $R_{\mathrm{OLS}}^{2}$. In all the cases examined, the values of $R_{\left(S_{\hat{\beta }}\right)}^{2}$ remain close to those of $R_{\mathrm{OLS}}^{2}$. It is worth noting that in this specific setting, $R_{\mathrm{MZ}}^{2}$ is also a good approximation to the OLS *R* ^2^, and it is comparable to our $R_{\left(S_{\hat{\beta }}\right)}^{2}$. But $R_{\mathrm{McF}}^{2}$ is much smaller than the OLS *R* ^2^.

**Setting 2** (p=20 with moderate and strong correlations). This simulation setting is similar to the previous one except that the set of explanatory variables is larger (p=20) and the correlations between certain variables are much stronger (e.g., Cor $\left(X_{1},X_{2}\right)=.7$). Specifically, we set $\alpha_{1}=1.2,\alpha_{2}=3.6$, and the coefficients {*β*_1_, *β*_3_, *β*_4_, *β*_11,_…,*β*_20_} = {.8, .6, .4, .5, …, .5}. The structure of this setting is illustrated below.


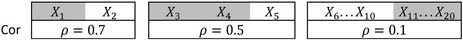


The four *R* ^2^s are compared in Table 3. The values of our $R_{\left(S_{\hat{\beta }}\right)}^{2}$ remain close to those of $R_{\mathrm{OLS}}^{2}$ in all the models considered, including the full model, the models excluding a single or all non‐signals, and the models excluding a single or several true signals. Once again, we observe that $R_{\mathrm{MZ}}^{2}$ approximates $R_{\mathrm{OLS}}^{2}$ well and that $R_{\mathrm{McF}}^{2}$ is consistently smaller.

**TABLE 3:** *R* ^2^ measures for probit models used in simulation setting 2

	Full model	Models w/o non‐signals		Models w/o true signals	
	$ℳ\left\{X_{1},\ldots ,X_{20}\right\}$	$ℳ\left\{-X_{2}\right\}$	$ℳ\left\{-\left(X_{6},\ldots ,X_{10}\right)\right\}$	$ℳ\left\{-X_{1}\right\}$	$ℳ\left\{-\left(X_{1},X_{3},X_{11}\right)\right\}$
n=500					
$R_{\mathrm{OLS}}^{2}$	.866	.865	.864	.819	.753
$R_{\left(S_{\beta }\right)}^{2}$	.866	.865	.864	.820	.753
$R_{\left(S_{\hat{\beta }}\right)}^{2}$	.876	.875	.872	.829	.761
$R_{\mathrm{MZ}}^{2}$	.873	.872	.870	.827	.760
$R_{\mathrm{McF}}^{2}$	.582	.581	.577	.517	.440
Accuracy	.792	.791	.789	.757	.713
n=1000					
$R_{\mathrm{OLS}}^{2}$	.863	.863	.862	.817	.750
$R_{\left(S_{\beta }\right)}^{2}$	.863	.863	.862	.817	.750
$R_{\left(S_{\hat{\beta }}\right)}^{2}$	.868	.868	.866	.822	.755
$R_{\mathrm{MZ}}^{2}$	.867	.866	.865	.821	.754
$R_{\mathrm{McF}}^{2}$	.572	.572	.570	.509	.433
Accuracy	.787	.786	.785	.752	.709

*Note*: Results presented are arithmetic averages over 2000 simulation repetitions. w/o = without.

**Setting 3** (p=4 with a strong correlation). To show the advantage of our surrogate $R_{\left(S\right)}^{2}$ over McKelvey and Zavoina's $R_{\mathrm{MZ}}^{2}$, we consider a setting similar to the seting of the counterexample in the introduction. The only difference is that we included two additional variables $X_{3}$ and $X_{4}$, both of which are independent of Y. This simulation setting is illustrated below.


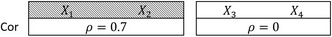


The results in Table 4 confirm the non‐monotonicity of $R_{\mathrm{MZ}}^{2}$. When n=1000, its value is .629 for the full mode, and it *increases* to .759 for a smaller model that does not contain $X_{2}$. As a result, it is twice as large as the value of $R_{\mathrm{OLS}}^{2}$. Such a notable increase is misleading in the practice of goodness‐of‐fit evaluation, and the disparity between $R_{\mathrm{MZ}}^{2}$ and $R_{\mathrm{OLS}}^{2}$ challenges the claim that the former is a good approximation to the latter. In contrast, Table 4 shows that our surrogate $R_{S_{\hat{\beta }}}^{2}$ maintains monotonicity between nested models, and its values remain close to those of $R_{\mathrm{OLS}}^{2}$. In short, it is the only goodness‐of‐fit measure that stays numerically close to $R_{\mathrm{OLS}}^{2}$ across the models considered here.

**TABLE 4:** *R* ^2^ measures for probit models used in simulation setting 3

	Full model	Models w/o non‐signals		Models w/o true signals	
	$ℳ\left\{X_{1},\ldots ,X_{4}\right\}$	$ℳ\left\{-X_{4}\right\}$	$ℳ\left\{-\left(X_{3},X_{4}\right)\right\}$	$ℳ\left\{-X_{2}\right\}$	$ℳ\left\{-\left(X_{1},\ldots ,X_{4}\right)\right\}$
n=1000					
$R_{\mathrm{OLS}}^{2}$	.608	.608	.607	.310	.000
$R_{\left(S_{\hat{\beta }}\right)}^{2}$	.630	.628	.625	.335	.000
$R_{\left(S_{\beta }\right)}^{2}$	.608	.608	.607	.310	.000
$R_{\mathrm{MZ}}^{2}$	.629	.627	.624	**.759**	.000
$R_{\mathrm{McF}}^{2}$	.537	.536	.535	.469	.000
AUC	.951	.951	.951	.945	.500
n=5000					
$R_{\mathrm{OLS}}^{2}$	.607	.607	.607	.309	.000
$R_{\left(S_{\hat{\beta }}\right)}^{2}$	.610	.610	.609	.313	.000
$R_{\left(S_{\beta }\right)}^{2}$	.607	.607	.607	.310	.000
$R_{\mathrm{MZ}}^{2}$	.610	.610	.609	**.753**	.000
$R_{\mathrm{McF}}^{2}$	.532	.531	.531	.463	.000
AUC	.950	.950	.950	.945	.500

*Note*: Results presented are arithmetic averages over 2000 simulation repetitions. w/o = without. Bold values is to highlight numerical results worth attention.

In what follows, we examine the performance of the interval *R* ^2^ measures. Presented in Table 5 are the lower and upper bounds (averaged over 1000 simulation repetitions) of 95% confidence intervals as well as the actual probabilities of those intervals containing the limit of the OLS *R* ^2^. We do not include $R_{\mathrm{McF}}^{2}$ as its limit is in theory different from that of the OLS *R* ^2^ (see our discussion in Section Discussion). Table 5 shows that McKelvey and Zavoina's interval tends to produce slightly lower coverage probabilities than ours across the models in settings 1 and 2 (e.g., 93.4% versus 95.3% for the full model in setting 1 when n=1000). More importantly, in setting 3, which is similar to the counterexample in the introduction, the non‐monotonicity issue of McKelvey and Zavoina's $R_{\mathrm{MZ}}^{2}$ causes it to break down when $X_{2}$ is excluded from the model. In this case, the bold numbers in the last column of Table 5 show that its coverage probability turns out to be zero. A close look of the result reveals that the average of the lower bound is .648, far greater than the the limit of $R_{\mathrm{OLS}}^{2}$, which is .309.

**TABLE 5:** Interval *R* ^2^ measures and their coverage probabilities

Setting 1	$ℳ\left\{X_{1},\ldots ,X_{10}\right\}$	$ℳ\left\{-X_{10}\right\}$	$ℳ\left\{-X_{1}\right\}$	$ℳ\left\{-\left(X_{1},X_{2}\right)\right\}$
n=1000				
$R_{\left(S_{\hat{\beta }}\right)}^{2}$	(.763, .830) (95.3%)	(.762, .829) (96.0%)	(.636, .724) (97.5%)	(.523, .625) (97.7%)
$R_{\mathrm{MZ}}^{2}$	(.765, .827) (93.4%)	(.764, .826) (93.9%)	(.638, .721) (96.6%)	(.524, .623) (96.8%)
n=2000				
$R_{\left(S_{\hat{\beta }}\right)}^{2}$	(.768, .817) (97.3%)	(.768, .816) (97.5%)	(.644, .707) (97.6%)	(.533, .606) (99.0%)
$R_{\mathrm{MZ}}^{2}$	(.770, .814) (95.9%)	(.770, .814) (96.0%)	(.646, .706) (96.4%)	(.534, .605) (98.8%)
Limit $R_{\mathrm{OLS}}^{2}$	.788	.788	.671	.565

Setting 2	$ℳ\left\{X_{1},\ldots ,X_{20}\right\}$	$ℳ\left\{-X_{2}\right\}$	$ℳ\left\{-X_{1}\right\}$	$ℳ\left\{-\left(X_{1},X_{3},X_{11}\right)\right\}$
n=1000				
$R_{\left(S_{\hat{\beta }}\right)}^{2}$	(.849, .897) (82.3%)	(.848, .896) (84.1%)	(.798, .857) (89.0%)	(.724, .797) (97.2%)
$R_{\mathrm{MZ}}^{2}$	(.849, .894) (81.6%)	(.849, .893) (83.4%)	(.799, .854) (86.9%)	(.726, .795) (96.0%)
n=2000				
$R_{\left(S_{\hat{\beta }}\right)}^{2}$	(.849, .884) (91.2%)	(.849, .884) (91.9%)	(.800, .842) (94.6%)	(.729, .780) (97.2%)
$R_{\mathrm{MZ}}^{2}$	(.850, .882) (90.3%)	(.849, .882) (91.0%)	(.801, .841) (91.9%)	(.730, .779) (95.5%)
Limit $R_{\mathrm{OLS}}^{2}$	.860	.860	.815	.748

Setting 3	$ℳ\left\{X_{1},\ldots ,X_{4}\right\}$	$ℳ\left\{-X_{4}\right\}$	$ℳ\left\{-\left(X_{3},X_{4}\right)\right\}$	$ℳ\left\{-X_{2}\right\}$
n=1000				
$R_{\left(S_{\hat{\beta }}\right)}^{2}$	(.568, .825) (86.2%)	(.564, .823) (88.9%)	(.559, .820) (91.5%)	(.216, .700) (90.5%)
$R_{\mathrm{MZ}}^{2}$	(.572, .822) (85.0%)	(.568, .820) (87.3%)	(.564, .817) (90.0%)	**(.648, .880) (.00%)**
n=2000				
$R_{\left(S_{\hat{\beta }}\right)}^{2}$	(.570, .697) (91.4%)	(.567, .694) (92.0%)	(.565, .692) (93.4%)	(.226, .486) (91.9%)
$R_{\mathrm{MZ}}^{2}$	(.573, .693) (89.4%)	(.570, .691) (90.9%)	(.568, .689) (91.8%)	**(.670, .845) (.00%)**
Limit $R_{\mathrm{OLS}}^{2}$	.607	.607	.607	.309

*Note*: Results are presented in the form (*a*, *b*) (*c*%), where *a* and *b* are the lower and upper bounds of the 95% confidence intervals, respectively, and *c*% is the actual coverage probability containing the limit of $R_{\mathrm{OLS}}^{2}$.

To summarize, the non‐monotonicity seen in Table 4 and the zero probability of covering the benchmark seen in Table 5 numerically prove that McKelvey and Zavoina's method is inherently defective in terms of approximating the OLS *R* ^2^. In contrast, the surrogate *R* ^2^ is consistently close to the OLS *R* ^2^, and its associated interval achieves a reasonable coverage probability (e.g., >90%) across all the models and settings considered when the sample size is moderately large (e.g., *n* = 2000). In Appendix S1, we present simulation results when the full model is misspecified under settings similar to those in this section. The numerical results demonstrate that our surrogate *R* ^2^ maintains monotonicity between nested models whereas McKelvey and Zavoina's *R* ^2^ does not.

## REAL DATA EXAMPLE

We illustrate the utility of the proposed surrogate $R_{\left(S\right)}^{2}$ in an actual model building procedure for a wine tasting preference data set. We stress that the focus is not on what criteria to select variables but on how to measure the goodness of fit of each candidate model considered in the entire model building process, and how to quantify and compare the contribution of each variable to the final model in terms of the explanatory power.

In the wine industry, it is of critical importance to understand how physicochemical properties may influence human tasting preference. To this end, Cortez et al. (2009) analysed a large survey (*n* = 4898) of vinho verde wine from the North of Portugal. The outcome of interest *preference* is a rating score with ordinal values between 0 and 10. The observed ratings are 3 (20 observations), 4 (163), 5 (1457), 6 (2198), 7 (880), 8 (175), and 9 (5), while other ratings are not present in the data set. The physicochemical properties examined in their study include acidity, sugar, dioxide, pH, and others (see Table 6 for a complete list). Including all of them as explanatory variables, we use the probit regression model (1) to initiate a full model. The results of the analysis are displayed in Table 6. By calling this full model into the *sure* package built for R (Greenwell et al., 2018), we simulate a sample of the surrogate response $S_{\text{preference}}$. Such a sample (*n* = 4898) will be used to evaluate the goodness of fit of a series of reduced models in the entire modelling process. We summarize in Table 7 our analysis performed in steps 0–3 as discussed below.

**TABLE 6:** An initial full‐model analysis of the wine quality data using a probit model

	Coefficient estimate	Standard error
*fixed. Acidity*	.092^***^	(.022)
*volatile. Acidity*	−2.804^***^	(.168)
*citric. Acid*	.018	(.139)
*residual. Sugar*	.116^***^	(.004)
*chlorides*	−.410	(.787)
*free.sulfur.dioxide*	.005^***^	(.001)
*total.sulfur.dioxide*	−.0004	(.001)
*density*	−209.996^***^	(.255)
*pH*	.973^***^	(.119)
*sulfates*	.905^***^	(.140)
*alcohol*	.290^***^	(.017)
Observations	4898	

${}^{*}p<.1$; ${}^{**}p<.05$; ${}^{***}p<.01$.

**TABLE 7:** *R* ^2^ measures for probit models trained from the wine tasting preference data

	$R_{\left(S_{\hat{\beta }}\right)}^{2}$	$\Delta \left(R_{\left(S_{\hat{\beta }}\right)}^{2}\right)$	${\mathrm{CI}}_{0.95}$	$R_{\mathrm{MZ}}^{2}$	$R_{\mathrm{McF}}^{2}$
Step 0. Full model	**.3210**		(.298, .346)	**.3186**	.1296
Step 1. Initial pruning					
$ℳ\left\{-\text{insignificant}\;\text{variables}\right\}$	**.3209**	.03%	(.297, .346)	**.3188**	.1295
Step 2. Eliminating collinearity					
$ℳ\left\{-\text{residual}.\text{sugar}\;\&\;\text{alcohol}\right\}$	.163	**49.21%**	(.138, .191)	.162	.061
$ℳ\left\{-\text{density}\right\}$	.310	3.40%	(.285, .332)	.306	.124
Step 3. Final ranking					
$ℳ\left\{-\text{alcohol}\right\}$	.070	**77.42%**	(.056, .088)	.070	.025
$ℳ\left\{-\text{volatile}.\text{acidity}\right\}$	.247	**20.32%**	(.224, .271)	.245	.096
$ℳ\left\{-\text{residual}.\text{sugar}\right\}$	.292	**5.81%**	(.267, .315)	.290	.116
$ℳ\left\{-\text{free}.\text{sulfur}.\text{dioxide}\right\}$	.305	1.61%	(.280, .327)	.303	.122
$ℳ\left\{-\text{sulfates}\right\}$	.307	.97%	(.282, .329)	.305	.123
$ℳ\left\{-\text{fixed}.\text{acidity}\right\}$	.307	.97%	(.282, .330)	.305	.123
$ℳ\left\{-\mathrm{pH}\right\}$	.309	.32%	(.284, .331)	.307	.124

*Note*: Bold values is to highlight numerical results worth attention.

Step 0: *Evaluate the goodness of fit of the full model*. Our surrogate *R* ^2^ value is .3210 for the full model. The result can be interpreted as a 32.10% reduction of the total variability of the *surrogate response* explained by the eleven explanatory variables. McKelvey and Zavoina's $R_{\mathrm{MZ}}^{2}=.3186$ is similar, whereas McFadden's $R_{\mathrm{McF}}^{2}=.1296$ is much lower.

Step 1: *Evaluate the goodness of fit of a reduced model excluding all insignificant variables*. As the variables *citric.acid, chlorides* and *total.sulfur.dioxide* are not significant, we remove them and refit a probit model. For this smaller model, we observe in Table 7 that our surrogate $R_{\left(S_{\hat{\beta }}\right)}^{2}$ exhibits a small amount of reduction of the goodness of fit (.03%). This negligible reduction suggests that these three variables contribute little to explaining the variability of the tasting preference. It is worth noting that $R_{\mathrm{MZ}}^{2}$ nevertheless becomes slightly larger (.3188) than its initial value (.3186) for the full model. This real data result confirms our discovery ‐ $R_{\mathrm{MZ}}^{2}$ cannot guarantee the monotonicity between nested models – and exemplifies the need for an *R* ^2^ measure that does maintain the monotonicity.

Step 2: *Decide which variables to keep in the presence of multicollinearity*. The variable *density* is highly correlated with *residual.sugar* and *alcohol*, with correlations of .84 and −.78, respectively. To decide which variables to remove, we fit two reduced models: one without *density* and the other without {*residual.sugar, alcohol*}. We see a small reduction of *R* ^2^ in the former model (3.40%) yet a much greater reduction in the latter (49.21%). Therefore, we decide to remove *density* as a remedy to avoid multicollinearity.

Step 3: *Rank the remaining variables based on their contribution to R* ^2^. We drop the remaining variables one at a time and examine the reduction in *R* ^2^. The top three variables are *alcohol* (77.42%), *volatile.acidity* (20.32%), and *residual.sugar* (5.81%). The ranking seen in Table 7 is consistent with the AIC‐ or BIC‐based ranking. The advantage of our *R* ^2^‐based ranking is that it allows the interpretation in terms of the explained variance, with the aid of the notion of the surrogate response. As a result, practitioners can read Table 7 as if *the response were continuous and the model were linear*. They can decide, based on their domain knowledge and specific needs, whether or not to further trim the model. For instance, the variable *pH*, sitting at the bottom of Table 7, may be removed for a more parsimonious model, considering that it contributes merely .32% to the *R* ^2^ value.

To conclude this section, we summarize the utility of our surrogate *R* ^2^ measure in this real data analysis. First, as a result of its similarity to $R_{\mathrm{OLS}}^{2}$ (C1) and its monotonicity (C3), the surrogate *R* ^2^ measure can guide the entire model building and trimming process in the same way as the OLS *R* ^2^ is used for linear models. Second, as it can be interpreted as the degree of the explained variation (C2), the surrogate *R* ^2^ allows us to evaluate the contribution of a single variable (or a group of variables) to explaining the total variability. Third, as our method maps the variability of the ordinal data (0–10 rating scales) onto the continuous scale of the latent outcome, it enables domain researchers to compare *R* ^2^ values across different samples and models. To elaborate on the last point, it is well known that wine experts traditionally use a 100‐point scoring system to rate wines, and numeric scores (e.g., 88, 93, …) are often seen in retail or wholesale stores such as Kroger and Costco. Other online marketplaces or smartphone apps, such as Vivino, invite users to rate wines using a five‐star system, which gives numeric scores (e.g., 3.8, 4.5, …). As our surrogate *R* ^2^ enables comparability across different samples and models, we can use the surrogate *R* ^2^ value obtained here, which is 32%, to benchmark the OLS *R* ^2^ values when analysing wine data sets from Kroger, Costco, or Vivino.

## DISCUSSION

In this paper we have proposed a new *R* ^2^ to measure the goodness of fit of a given probit model. As the explained proportion of the variance of the surrogate response, it is conceptually simple and computationally straightforward. We have justified both theoretically and numerically that the surrogate *R* ^2^ approximates the OLS *R* ^2^ if the latent continuous outcome can be observed. It solves the serious non‐monotonicity issue observed in McKelvey and Zavoina's $R_{\mathrm{MZ}}^{2}$. To conclude, our surrogate *R* ^2^ is the only one known so far that meets all three crucial criteria (C1)–(C3) discussed in the introduction. It therefore makes it legitimate to compare *R* ^2^ values of different empirical models across multiple samples, regardless of whether the response type is continuous, binary, or ordinal. An R package *SurrogateRsq* will be made available to facilitate the numerical implementation in practice.

We conclude with further discussions on the implementation of our method.

### Using the full model to generate a common surrogate response for smaller models

In practice, data analysts may need to compare *R* ^2^ values of several probit models, nested or non‐nested, trained for the same data set. In order to use our method, data analysts should initiate a proper full model, possibly using domain knowledge as well as statistical tools such as variable selection and model diagnostics. This full model should be used to generate a single surrogate response S that will be used across the board to calculate surrogate *R* ^2^ values for any smaller model. To consider an illustrative example, if $X_{1}$ and $X_{2}$ are deemed potentially ‘important’ and it is found to be necessary to include the square of $X_{1}$, we should set the full model as (F) $Y∼1+X_{1}+X_{2}+X_{1}^{2}$ and use it to generate a surrogate response S. This response should be used to calculate surrogate *R* ^2^ values for smaller models such as (A) $Y∼1+X_{1}$, (B) $Y∼1+X_{1}+X_{1}^{2}$, or (C) $Y∼1+X_{1}+X_{2}$. To make this clearer, data analysts should not use models (A), (B), and (C) to separately generate three surrogate responses and calculate their corresponding *R* ^2^ values. This method is flawed. It will yield a measure similar to McKelvey and Zavoina's *R* ^2^, and thus it cannot achieve monotonicity and maintain a good approximation to the OLS *R* ^2^.

It is not a strong requirement to have a common (surrogate) response for the purpose of calculating and comparing *R* ^2^ for a group of models. In fact, it is seen in linear regression analysis, where a single continuous response is readily available – so there is no need to simulate it – and this common response is used to calculate *R* ^2^ for any smaller models. Monotonicity is simply a mathematical result of having a common *Y*. Following the same line of thought, our method seeks a common continuous ‘*Y*’ for a group of smaller probit models by simulating it from the full probit model. It is this similarity that allows us to establish the theoretical and numerical results presented in this paper.

### Dependence of the surrogate *R* ^2^ on a full model

The surrogate *R* ^2^ depends on the specification of the full model. If domain researchers have acquired data of new explanatory variables and decided to add some of them to the full model, the proposed procedure needs to be rerun to adjust our *R* ^2^. The dependence on the full model is not necessarily a weakness as long as we acknowledge that *inference is always made conditional on the variables available to us*. As for conditional inference, the result certainly depends on what is conditioned (e.g., the full model in the case discussed here). When new variables become available, conditional inference should be updated and the result may not remain the same. From the perspective of conditional inference, it may be an advantage of our surrogate method *not* always maintaining

$$R^{2}\left\{X_{1},\ldots ,X_{p}|X_{1},\ldots ,X_{p},X_{p+1}\right\}=R^{2}\left\{X_{1},\ldots ,X_{p}|X_{1},\ldots ,X_{p}\right\},$$

where $R^{2}\left\{X_{1},\ldots ,X_{p}|X_{1},\ldots ,X_{p},X_{p+1}\right\}$ is the *R* ^2^ of the model containing $\left\{X_{1},\ldots ,X_{p}\right\}$ when the set of variables $\left\{X_{1},\ldots ,X_{p},X_{p+1}\right\}$ is available.

To explain the reason, we restate this discrete data question in the missing‐data framework: how can we uncover the underlying OLS *R* ^2^ when the continuous data of Z are missing and we only observe whether or not Z is located in an interval? Through the lens of missing‐data inference, we would not expect an inference outcome, such as *R* ^2^, to remain the same regardless of how much information is given: a smaller set of variables $\left\{X_{1},\ldots ,X_{p}\right\}$ or a larger set $\left\{X_{1},\ldots ,X_{p},X_{p+1}\right\}$. In fact, once a larger set of information becomes available. we should rerun the analysis as a more accurate inference may be achieved with the addition of a new variable $X_{p+1}$. In fact, $R^{2}\left\{X_{1},\ldots ,X_{p}|X_{1},\ldots ,X_{p},X_{p+1}\right\}$ can be viewed as an update of $R^{2}\left\{X_{1},\ldots ,X_{p}|X_{1},\ldots ,X_{p}\right\}$ with the added new information contained in the variable $X_{p+1}$. This update may correct McKelvey and Zavoina's method when the non‐monotonicity is an issue. This point is seen in our simulation and real‐data studies.

This line of thinking leads to an *R* ^2^ that is asymptotically equivalent to the surrogate *R* ^2^ and corrects McKelvey and Zavoina's *R* ^2^ for reduced models. Suppose a full model is identified with $X_{0}$ being the centered design matrix as stated in Section Resemblance to the OLS *R* ^2^. For a reduced model, we let $X_{r}$ be a submatrix containing the columns of $X_{0}$ that correspond to the selected variables. An asymptotic version of our surrogate *R* ^2^ is

$$R_{\left(S\right),\text{asym}}^{2}=\frac{{\hat{\beta }}^{\prime }X_{0}^{\prime }X_{r}{\left(X_{r}^{\prime }X_{r}\right)}^{-1}X_{r}^{\prime }X_{0}\hat{\beta }}{{\hat{\beta }}^{\prime }X_{0}^{\prime }X_{0}\hat{\beta }+n},$$

where $\hat{\beta }$ is the same coefficient estimate trained from the full model. When applied to the full model itself (i.e., $X_{r}=X_{0}$), $R_{\left(S\right),\text{asym}}^{2}={\hat{\beta }}^{\prime }X_{0}^{\prime }X_{0}\hat{\beta }/\left({\hat{\beta }}^{\prime }X_{0}^{\prime }X_{0}\hat{\beta }+n\right)=R_{\mathrm{MZ}}^{2}$. This result verifies again that for the full model, our surrogate *R* ^2^ is asymptotically equivalent to McKelvey and Zavoina's *R* ^2^ (see also Proposition 2). The formula for $R_{\left(S\right),\text{asym}}^{2}$ explains in a mathematical expression why our surrogate *R* ^2^ may differ from McKelvey and Zavoina's *R* ^2^ for reduced models and how the correction should be made to maintain monotonicity.

### Using the *R* ^2^ measure strategically with variable selection and model diagnostics

Statistical techniques, such as variable selection, model diagnostics, and the *R* ^2^ measure proposed here, cannot substitute for one another. They should be used strategically to build a useful model and deepen the understanding of the subject matter. Given a data set, we would recommend carrying out a three‐step procedure as follows.

Step 1. Use AIC, BIC, the lasso or any other variable selection criterion deemed appropriate to trim a set of explanatory variables to a ‘manageable’ size (e.g., <20).

Step 2. Use model diagnostic tools, such as those in Liu and Zhang (2018), to adjust the form of the model and add necessary elements (e.g., higher‐order and interaction terms).

Step 3. Initiate a full model based on steps 1–2 and simulate a surrogate response. Use this surrogate response to: (i) calculate *R* ^2^ for the full model, which value can be used to compare with *R* ^2^ obtained from other empirical samples and models on the same subject matter; (ii) calculate *R* ^2^ values for any other smaller models so as to facilitate model comparison for the present sample and project; (iii) gauge the *R* ^2^ contribution of each individual variable to the overall explanatory power so as to understand its importance to the subject issue. Points (i)–(iii) are illustrated in our real data analysis, which exemplifies that the role of *R* ^2^ measure cannot be substituted by variable selection or model diagnostics.

### Variance‐based versus entropy‐based *R* ^2^ measures

The OLS *R* ^2^ examines the uncertainty reduction using the ratio between the explained variance and the total variance of the response variable. Our proposed surrogate *R* ^2^ aims to approximate it when probit models are applied to discrete data. However, variance is not the only measure of uncertainty, and Shannon entropy is clearly an alternative. Note that McFadden's *R* ^2^ is the ratio of two likelihood functions which approach the conditional (on X) and unconditional entropies when the sample size increases. It is therefore another reasonable *R* ^2^ measure to use in practice. As it only relies on likelihood functions, the merit of McFadden's *R* ^2^ lies in its applicability to a wide range of regression models, including the probit models considered in this paper. But for probit models specifically, our surrogate *R* ^2^ is shown to be analogous to the OLS *R* ^2^, which is a property needed in empirical analysis to achieve comparability between different models and studies. The idea can be naturally extended to other latent variable models, such as logistic regression models. Our preliminary investigation has shown that the *R* ^2^ defined in a similar way for logistic models also meets criteria (C1)–(C3). In the context of bankruptcy studies, it allows us to compare financial markets in different countries. The research results will be reported elsewhere. As a closing remark, we point out that a further development of the notion of *R* ^2^ for general models without any latent variable structure is of much greater interest.

## CONFLICT OF INTEREST

All authors declare no conflict of interest.

## Supporting information


Appendix S1.

## Data Availability

The data used in this paper are available in the the UC Irvine Machine Learning Repository (http://archive.ics.uci.edu/ml/datasets/Wine+Quality).
