# Supplementary material for: A new goodness‐of‐fit measure for probit models: Surrogate R 2
Source: Br J Math Stat Psychol. 2022 Oct 17;76(1):192–210. doi: 10.1111/bmsp.12289 (PMC10092347; doi:10.1111/bmsp.12289)
Supplement: Supplementary file 1 — Appendix S1. [file BMSP-76-192-s001.pdf]

Supplementary Materials

A. Technical details for Section 3

Proof for Proposition 1. In Section 3, we have established

$$R^2_{OLS} = \frac{\parallel \boldsymbol{X}_0 \hat{\boldsymbol{\beta}}^{(\boldsymbol{z})} \parallel^2}{\parallel \boldsymbol{X}_0 \hat{\boldsymbol{\beta}}^{(\boldsymbol{z})} \parallel^2 + \parallel P_{V_{\boldsymbol{x}}^{\perp}} \boldsymbol{z} \parallel^2}.$$

Given that (i) $\hat{\boldsymbol{\beta}}^{(\boldsymbol{z})} \sim N(\boldsymbol{\beta}, (\boldsymbol{X}_0'\boldsymbol{X}_0)^{-1})$, (ii) $\parallel P_{V_{\boldsymbol{x}}^{\perp}} \boldsymbol{z} \parallel^2 \sim \chi^2_{(n-p-1)}$ and (iii) $\hat{\boldsymbol{\beta}}^{(\boldsymbol{z})}$ and $\parallel P_{V_{\boldsymbol{x}}^{\perp}} \boldsymbol{z} \parallel^2$ are independent (Stapleton, 1995, p.81), we let two independent random vectors

$$\boldsymbol{u} \sim N(\boldsymbol{X_0}\boldsymbol{\beta}, \boldsymbol{X_0}(\boldsymbol{X}_0'\boldsymbol{X}_0)^{-1}\boldsymbol{X}_0') \text{ and } v \sim \chi^2_{(n-p-1)}.$$

Therefore, R^2_{OLS} follows the same distribution as the variable $\{\parallel u \parallel^2 / (\parallel u \parallel^2 + v)\}$.

Using a sample of the surrogate response $\boldsymbol{s} = (s_1, \ldots, s_n)'$, we also have established

$$R^2_{(S)} = \frac{\parallel \boldsymbol{X}_0 \hat{\boldsymbol{\beta}}^{(\boldsymbol{s})} \parallel^2}{\parallel \boldsymbol{X}_0 \hat{\boldsymbol{\beta}}^{(\boldsymbol{s})} \parallel^2 + \parallel P_{V_{\boldsymbol{x}}^{\perp}} \boldsymbol{s} \parallel^2},$$

and (i) $\hat{\boldsymbol{\beta}}^{(\boldsymbol{s})} \sim N(\boldsymbol{\beta}, (\boldsymbol{X}_0'\boldsymbol{X}_0)^{-1})$, (ii) $\parallel P_{V_{\boldsymbol{x}}^{\perp}} \boldsymbol{s} \parallel^2 \sim \chi^2_{(n-p-1)}$ and (iii) $\hat{\boldsymbol{\beta}}^{(\boldsymbol{s})}$ and $\parallel P_{V_{\boldsymbol{x}}^{\perp}} \boldsymbol{s} \parallel^2$ are independent. Following the same arguments for R^2_{OLS}, we immediately have the result that $R^2_{(S)}$ follows the same distribution as the variable $\{\parallel u \parallel^2 / (\parallel u \parallel^2 + v)\}$. Therefore, $R^2_{(S)}$ follows the same distribution as that of R^2_{OLS}. This completes the proof. □

Proof for Proposition 2. We write

$$\begin{aligned} R^2_{OLS} &= \frac{\parallel \boldsymbol{X}_0 \hat{\boldsymbol{\beta}}^{(\boldsymbol{z})} \parallel^2}{\parallel \boldsymbol{X}_0 \hat{\boldsymbol{\beta}}^{(\boldsymbol{z})} \parallel^2 + \parallel P_{V_{\boldsymbol{x}}^{\perp}} \boldsymbol{z} \parallel^2} \\ &= \frac{\hat{\boldsymbol{\beta}}^{(\boldsymbol{z})'} \boldsymbol{X}_0' \boldsymbol{X}_0 \hat{\boldsymbol{\beta}}^{(\boldsymbol{z})}}{\hat{\boldsymbol{\beta}}^{(\boldsymbol{z})'} \boldsymbol{X}_0' \boldsymbol{X}_0 \hat{\boldsymbol{\beta}}^{(\boldsymbol{z})} + \parallel P_{V_{\boldsymbol{x}}^{\perp}} \boldsymbol{z} \parallel^2} \\ &= \frac{\hat{\boldsymbol{\beta}}^{(\boldsymbol{z})'} \boldsymbol{X}_0' \boldsymbol{X}_0 \hat{\boldsymbol{\beta}}^{(\boldsymbol{z})} / (n-1)}{(\hat{\boldsymbol{\beta}}^{(\boldsymbol{z})'} \boldsymbol{X}_0' \boldsymbol{X}_0 \hat{\boldsymbol{\beta}}^{(\boldsymbol{z})} + \parallel P_{V_{\boldsymbol{x}}^{\perp}} \boldsymbol{z} \parallel^2)/(n-1)}. \end{aligned}$$

From the proof of Proposition 1, we have indicated that (i) $\hat{\boldsymbol{\beta}}^{(\boldsymbol{z})} \sim N(\boldsymbol{\beta}, (\boldsymbol{X}_0'\boldsymbol{X}_0)^{-1})$ and (ii) $\parallel P_{V_{\boldsymbol{x}}^{\perp}} \boldsymbol{z} \parallel^2 \sim \chi^2_{(n-p-1)}$. As the sample size $n \to \infty$, it is known that $\hat{\boldsymbol{\beta}}^{(\boldsymbol{z})} \to \boldsymbol{\beta}$, $\parallel P_{V_{\boldsymbol{x}}^{\perp}} \boldsymbol{z} \parallel^2$

$/(n-p-1) \to 1$, and $\boldsymbol{X}_0'\boldsymbol{X}_0/n \to Cov(\boldsymbol{X}) = \boldsymbol{\Sigma}_x$ in probability. Therefore, R^2_{OLS} converges in probability to

$$\frac{\boldsymbol{\beta}'\boldsymbol{\Sigma}_x\boldsymbol{\beta}}{\boldsymbol{\beta}'\boldsymbol{\Sigma}_x\boldsymbol{\beta}+1}.$$

Similarly, following the same arguments for $R^2_{(S)}$, we can establish the result that $R^2_{(S)}$ converges to the same value in probability. This completes the proof. □

Proof for Proposition 3. The sample Pearson correlation coefficient between $\boldsymbol{s}$ and $\hat{\boldsymbol{s}}$ is

$$\begin{aligned} r_{\boldsymbol{s}\hat{\boldsymbol{s}}} &= \frac{(\boldsymbol{s},\hat{\boldsymbol{s}})}{\parallel \boldsymbol{s} \parallel\parallel \hat{\boldsymbol{s}} \parallel} = \frac{(\boldsymbol{s}-\mathbf{1}\bar{s},\hat{\boldsymbol{s}}-\mathbf{1}\bar{s})}{\parallel \boldsymbol{s}-\mathbf{1}\bar{s} \parallel\parallel \hat{\boldsymbol{s}}-\mathbf{1}\bar{s} \parallel} = \frac{(\boldsymbol{s}-\hat{\boldsymbol{s}}+\hat{\boldsymbol{s}}-\mathbf{1}\bar{s},\hat{\boldsymbol{s}}-\mathbf{1}\bar{s})}{\parallel \boldsymbol{s}-\mathbf{1}\bar{s} \parallel\parallel \hat{\boldsymbol{s}}-\mathbf{1}\bar{s} \parallel} \\ &= \frac{\parallel \hat{\boldsymbol{s}}-\mathbf{1}\bar{s} \parallel^2}{\parallel \boldsymbol{s}-\mathbf{1}\bar{s} \parallel\parallel \hat{\boldsymbol{s}}-\mathbf{1}\bar{s} \parallel} \\ &= \frac{\parallel \hat{\boldsymbol{s}}-\mathbf{1}\bar{s} \parallel}{\parallel \boldsymbol{s}-\mathbf{1}\bar{s} \parallel} \end{aligned}$$

with the $(\cdot,\cdot)$ denotes the inner product and $(\boldsymbol{s}-\hat{\boldsymbol{s}}) \perp (\hat{\boldsymbol{s}}-\mathbf{1}\bar{s})$. On the other hand,

$$R^2_{(S)} = \frac{SSR_{(S)}}{SST_{(S)}} = \frac{\parallel \hat{\boldsymbol{s}}-\mathbf{1}\bar{s} \parallel^2}{\parallel \boldsymbol{s}-\mathbf{1}\bar{s} \parallel^2}.$$

Therefore, $R^2_{(S)} = r^2_{\boldsymbol{s}\hat{\boldsymbol{s}}}$. This completes the proof. □

Proof for Proposition 4. By plugging in the formula of

$$F_{(S)} = \frac{\parallel \hat{\boldsymbol{s}}-\mathbf{1}\bar{s} \parallel^2/p}{\parallel \boldsymbol{s}-\hat{\boldsymbol{s}} \parallel^2/(n-p-1)},$$

we use simple algebra to derive

$$\begin{aligned} \frac{pF_{(S)}}{pF_{(S)}+(n-p-1)} &= \frac{\frac{(n-p-1)\|\hat{\boldsymbol{s}}-\mathbf{1}\bar{s}\|^2}{\|\boldsymbol{s}-\hat{\boldsymbol{s}}\|^2}}{\frac{(n-p-1)\|\hat{\boldsymbol{s}}-\mathbf{1}\bar{s}\|^2}{\|\boldsymbol{s}-\hat{\boldsymbol{s}}\|^2}+(n-p-1)} \\ &= \frac{\parallel \hat{\boldsymbol{s}}-\mathbf{1}\bar{s} \parallel^2}{\parallel \hat{\boldsymbol{s}}-\mathbf{1}\bar{s} \parallel^2 + \parallel \boldsymbol{s}-\hat{\boldsymbol{s}} \parallel^2} \\ &= \frac{\parallel \hat{\boldsymbol{s}}-\mathbf{1}\bar{s} \parallel^2}{\parallel \boldsymbol{s}-\mathbf{1}\bar{s} \parallel^2} \\ &= R^2_{(S)}. \end{aligned}$$

This completes the proof. □

Proof for Proposition 5. We let $V_{q1} = \mathcal{L}(\mathbf{1}, \boldsymbol{x}_1, \ldots, \boldsymbol{x}_{q1})$ be the linear space spanned by $\{\mathbf{1}, \boldsymbol{x}_1, \ldots, \boldsymbol{x}_{q1}\}$. Similarly, we let $V_{q2} = \mathcal{L}(\mathbf{1}, \boldsymbol{x}_1, \ldots, \boldsymbol{x}_{q2})$ and $V_{\mathbf{1}} = \mathcal{L}(\mathbf{1})$. We also have

$$R^2_{(S)}\{X_1, \ldots, X_{q1}\} = \frac{\| P_{V_{q1}} \boldsymbol{s} \|^2 - \| P_{V_{\mathbf{1}}} \boldsymbol{s} \|^2}{\| P_{V_{\mathbf{1}}^{\perp}} \boldsymbol{s} \|^2}$$

and

$$R^2_{(S)}\{X_1, \ldots, X_{q2}\} = \frac{\| P_{V_{q2}} \boldsymbol{s} \|^2 - \| P_{V_{\mathbf{1}}} \boldsymbol{s} \|^2}{\| P_{V_{\mathbf{1}}^{\perp}} \boldsymbol{s} \|^2}.$$

Since V_{q1} is a subspace of V_{q2}, we have $\| P_{V_{q1}} \boldsymbol{s} \|^2 \leq \| P_{V_{q2}} \boldsymbol{s} \|^2$. Therefore,

$$R^2_{(S)}\{X_1, \ldots, X_{q1}\} \leq R^2_{(S)}\{X_1, \ldots, X_{q2}\}.$$

This completes the proof. □

B. Additional simulations for Section 5

We conduct additional simulation studies to verify that our surrogate R^2 maintains monotonicity even when the full model is not specified correctly. To facilitate comparisons, we use the same settings as in Section 5 but specify full models incorrectly. Specifically,

- In Setting 1, we drop two true signals X_4 and X_5 and use the rest to build a full model. The corresponding R^2 measures are displayed in Table 8.
- In Setting 2, we drop two true signals X_1 and X_4 and use the rest to build a full model. The results are shown in Table 9.
- In Setting 3, we generate another variable X_5 which is correlated with X_1 ($\rho = 0.7$) but is independent of Y. We use this non-signal X_5, in place of the true signal X_1, and other variables to build a full model. The results are shown in Table 10.

The results in Tables 8-10 show that our proposed R^2 maintains monotonicity across all the settings when the full models are not specified correctly. In contrast, McKelvey-Zavoina's R^2_{MZ} exhibits non-monotonicity under Setting 3. For example, $R^2_{MZ} = 0.358$ for the full model, whereas $R^2_{MZ} = 0.362$ (increased!) for the reduced model $\mathcal{M}\{-X_2\}$.

Table 8: R^2 measures for misspecified probit models under simulation setting 1

	Misspecified full model	Models w/o non-signals		Models w/o true signals	
	$\mathcal{M}^*$	$\mathcal{M}^*\{-X_{10}\}$	$\mathcal{M}^*\{-(X_6 \ldots X_{10})\}$	$\mathcal{M}^*\{-X_1\}$	$\mathcal{M}^*\{-(X_1, X_2)\}$
$n = 200$					
$R^2_{(S_{\hat{\beta}})}$	0.690	0.686	0.663	0.541	0.397
R^2_{MZ}	0.684	0.681	0.661	0.537	0.394
R^2_{McFadden}	0.368	0.365	0.348	0.258	0.173
$n = 500$					
$R^2_{(S_{\hat{\beta}})}$	0.678	0.676	0.660	0.530	0.387
R^2_{MZ}	0.676	0.673	0.659	0.528	0.385
R^2_{McFadden}	0.360	0.358	0.346	0.251	0.168

Note: Results presented are the arithmetic averages over 2000 simulation repetitions.
*The misspecified full model does not contain X_4 and X_5 which are used to generate the response.

Table 9: R^2 measures for misspecified probit models under simulation setting 2

	Misspecified full Model	Models w/o non-signals		Models w/o true signals	
	$\mathcal{M}^*$	$\mathcal{M}^*\{-X_2\}$	$\mathcal{M}^*\{-(X_6 \ldots X_{10})\}$	$\mathcal{M}^*\{-X_3\}$	$\mathcal{M}^*\{-(X_3, X_{11})\}$
$n = 500$					
$R^2_{(S_{\hat{\beta}})}$	0.814	0.769	0.811	0.757	0.723
R^2_{MZ}	0.811	0.767	0.808	0.754	0.721
R^2_{McFadden}	0.497	0.447	0.494	0.434	0.401
$n = 1000$					
$R^2_{(S_{\hat{\beta}})}$	0.807	0.763	0.805	0.750	0.717
R^2_{MZ}	0.805	0.761	0.804	0.749	0.716
R^2_{McFadden}	0.490	0.441	0.489	0.428	0.396

Note: Results presented are the arithmetic averages over 2000 simulation repetitions.
*The misspecified full model does not contain X_1 and X_4 which are used to generate the response.

Table 10: R^2 measures for misspecified probit models under simulation setting 3

	Misspecified full model	Models w/o non-signals		Models w/o true signals	
	$\mathcal{M}^*$	$\mathcal{M}^*\{-X_4\}$	$\mathcal{M}^*\{-(X_3, X_4)\}$	$\mathcal{M}^*\{-X_2\}$	$\mathcal{M}^*\{-(X_2 \ldots X_5)\}$
$n = 1000$					
$R^2_{(S_{\hat{\beta}})}$	0.366	0.363	0.361	0.341	0.000
R^2_{MZ}	0.364	0.362	0.360	**0.367**	0.000
R^2_{McFadden}	0.213	0.211	0.210	0.202	0.000
$n = 5000$					
$R^2_{(S_{\hat{\beta}})}$	0.359	0.358	0.357	0.335	0.000
R^2_{MZ}	0.358	0.358	0.357	**0.362**	0.000
R^2_{McFadden}	0.209	0.209	0.209	0.199	0.000

Note: Results presented are the arithmetic averages over 2000 simulation repetitions.
*The misspecified full model does not contain X_1 which are used to generate the response but include a non-signal X_5 which is strongly correlated with X_1.
